# Can Combination Therapy of Conventional and Oriental Medicine Improve Poststroke Aphasia? Comparative, Observational, Pragmatic Study

**DOI:** 10.1155/2012/654604

**Published:** 2012-08-27

**Authors:** WooSang Jung, SeungWon Kwon, SeongUk Park, SangKwan Moon

**Affiliations:** ^1^Department of Cardiovascular and Neurologic Diseases, Kyung Hee University Oriental Medicine Hospital, 1 Hoegi-dong, Dongdaemun-gu, Seoul 130-702, Republic of Korea; ^2^Department of Cardiovascular and Neurologic Diseases, Kyung Hee University Hospital at Gangdong, Republic of Korea

## Abstract

The aim of the present study was to determine the effectiveness of oriental medicine therapy on poststroke aphasia. The outcome was measured as the delta value of the Aphasic Quotient score. Patients completed test at two timepoints: baseline and discharge time. Patients who received conventional therapy and language therapy were grouped in the Only Language Therapy group. Patients who received conventional therapy, language therapy, and an Oriental medicine regimen were grouped in the Combined oriental Medicine Therapy group. We compared the delta value of the Aphasic Quotient score between two groups. The Combined Oriental Medicine group exhibited a greater improvement than the Only Language Therapy group in the total Aphasic Quotient score and most subsection scores. In particular, there were statistically significant differences in total Aphasic Quotient score and subsections such as spontaneous speech, content delivery, comprehension, auditory verbal comprehension, and command performance. Among severe aphasic patients, the improvement of the Combined oriental Medicine group was better than that of the Only Language Therapy group. Through this study, we suggest combination therapy with the administration of oriental medicine and language therapy can be helpful in the treatment of post-stroke aphasic patients.

## 1. Introduction

Approximately 38% of stroke patients suffer from aphasia. Among them, 50% have aphasia with long-term disabilities after 6 months [1]. Recovery from aphasia is commonly achieved within 6 months after the onset of stroke. However, post-stroke aphasia can become a permanent deficit after 6 months. Therefore, post-stroke aphasia must be treated effectively within the first 6 months after the stroke. Some studies suggest that the duration of an effective aphasia treatment regimen should be 2–6 months [2–7]. 

Donepezil [8], bromocriptine,amphetamine, and piracetam [9, 10] are generally suggested for the treatment of aphasia. However, these drugs are not always prescribed because there is a lack of evidence concerning their overall effect on aphasia. Compared with conventional therapy strategies for the treatment of aphasia, Oriental medicine doctors have used several different modalities to treat this disease. Some case reports suggested that herbal complexes such as *jihwangeumja *[11–13], *cheongshinhaeeo-tang *[14], *seonghyangjeongkisan *[13],* cheongshindodam-tang *[13], and others [15] are effective in the treatment of aphasia. Other reports describe the use of “*scalp acupucnture*” a form of acupuncture, to heal aphasia [16, 17]. However, these reports were based on case studies. Furthermore, these studies did not include a control group. 

The aim of the present study was to determine the effectiveness of oriental medicine (consisting of herbal medicine, acupuncture, and moxibustion) on post-stroke aphasia by comparing the Aphasia Quotient (AQ) scores between a combined Oriental medicine therapy group and a non-Oriental medicine therapy group. Present study was performed as a pragmatic method understanding character of Oriental medicine, reflecting individuation of patients' general condition and symptoms.

## 2. Methods

### 2.1. Participants

The Kyung Hee University Oriental Medicine Hospital Institutional Review Board approved the present study (KOMC IRB 2009-14), which was conducted as a comparative, observational, and pragmatic study. Over a 31-month period spanning from March 2009 to September 2011, 77 Korean Asian patients in total (mean ± SD age 53.7  ± 14.4 years) with a clinical diagnosis of post-stroke aphasia of different severities (mild to severe) were enrolled in the study. Participants who were treated with language therapy after 6 months from the onset of stroke were also excluded from the study.

### 2.2. Study Design (Figure 1)

After admission to Kyung Hee Medical Center (Department of Cardiovascular and Neurologic Diseases of the Oriental Medicine Hospital, and Department of Neurology, Neurosurgery, and Rehabilitation of the Western Medicine Hospital), the participants received language therapy until they were discharged. The frequency and intensity of language therapy were adjusted individually according to the severity and the type of the participants' aphasia. Participants completed the K-WAB (Korean version of the Western Aphasia Battery) AQ test (Table 7) at 2 time-points: an initial (“baseline”) test was administered at the time of recruitment of the participants, and a second test was administered after the last language therapy session (discharge time). Discharge time was decided according to the Korean health policy which limit the admission duration of one hospital to 6–8 weeks. The AQ test was performed by four language therapists. All participants were evaluated by language therapist in charge twice. These language therapists did not know that they were participating in the present study. Calculation of the AQ test scores included the total AQ score and the AQ subsection score. The type of stroke was determined by the results of the initial AQ test. Medical records of each participant's age, medical history, and associated symptoms were recorded. The patients were diagnosed with stroke when neurological deficits were accompanied by abnormal findings in computed tomography (CT) or magnetic resonance imaging (MRI) of the brain. 

The participants were assigned to one of the two treatment groups. The Combined Oriental Medicine Therapy (COT) group admitted to the Oriental Medicine Hospital of Kyung Hee Medical Center and received conventional therapy that included taking antiplatelet agent, control risk factor (e.g. hypertension, diabetes mellitus, dyslipidemia and cardiac disease), rehabilitation exercise, working exercise for stroke, language therapy for aphasia, and an oriental medicine regimen that included herbal medicine, acupuncture, and moxibustion. oriental medicine therapy administered to patients in the COT group was adjusted individually according to the severity and type of aphasia, general condition, and associated symptoms. Details of the Oriental medicine therapies (herbal medicine type and contents, using frequency, acupuncture, method, and moxibustion method) were recorded in Table 1. Herbal medicine adjusted in each patient was selected by patient's condition, characters, and oriental diagnosis (pattern recognition). Acupuncture and moxibustion were conducted equally in all COT group patients. The Only Language Therapy (OLT) group was admitted to the Western Medicine Hospital of Kyung Hee Medical Center and received conventional therapy for stroke and language therapy for aphasia. In Kyung Hee Medical Center, there are two hospitals, Western Medicine Hospital which treat patients with conventional therapy and Oriental Medicine Hospital which treat patients with conventional therapy and oriental medicine therapy. Each patient who is admited Kyung Hee Medical Center can choose one of the two hospitals according to their preference. Therefore, inclusion in each group was not randomized, but based on the participants' choice.

### 2.3. Measurements

The AQ test was used to estimate the effectiveness of the oriental medicine combination therapy for aphasia. The primary outcome of this study was measured as the delta value of the total AQ score (difference between the initial total AQ score and the final total AQ score, “final total AQ score—initial total AQ score”). The secondary outcome was the delta value of each subsection score of the AQ test, such as spontaneous speech, comprehension, repetition, and naming. The third outcome was the change of aphasia severity distribution. Patients were classified into two severity groups according to the AQ scores as follows: non-severe aphasia: AQ 36.3 ~ 92.2, severe aphasia: AQ 0–36.2. We compared the scores of AQ test and the distribution of severity (severe, and non-severe) of the COT group with the OLT group.

### 2.4. Statistical Analysis

All data and results were analyzed using SPSS 12.0 for Windows. Fisher's exact test was used to compare categorical variables, including gender, handedness, type of stroke, type of aphasia, risk factors, frequency of related symptoms, and distribution of aphasia severity between the two groups. The Mann-Whitney *U* test was used to compare continuous variables such as age, education, treatment periods from onset, duration of treatment, and differences in AQ scores between the 2 groups. The Wilcoxon-signed rank test was performed to compare changes of AQ scores in each group. The McNemar test was performed to compare changes of the distribution of severity of aphasia.

## 3. Results

### 3.1. Baseline Assessment

Among the 77 participants, 47 were included in the COT group and 30 in the OLT group. However, the baseline characteristics of the two groups exhibited no significant differences with regard to gender, age, education, handedness, treatment period from onset, and duration of language therapy. There was no significant difference in stroke risk factors such as hypertension, diabetes mellitus, dyslipidemia, and heart disease (Table 2).

The total AQ score was 28.1 ± 25.6 in the COT group and 26.7 ± 26.2 in the OLT group. There were small differences in the total score between the 2 groups, which were not statistically significant. The subsection scores such as spontaneous speech, comprehension, repetition, and naming (naming things, control word association, sentence completion, and answers) demonstrated no statistically significant differences either (Table 2).

### 3.2. Type of Stroke

In the COT group, there were 30 infarction patients and 17 hemorrhage patients. In the OLT group, there were 14 infarction patients and 16 hemorrhage patients. The proportion of cerebral infarction subtypes based on the TOAST (Trials of Org 10172 in Acute Stroke Treatment) classification was not significantly different between the two groups (Table 3), nor was the proportion of cerebral hemorrhage subtypes (Table 3).

### 3.3. Type of Aphasia, and Related Symptoms

Twenty-five patients in the COT group and 16 patients in the OLT group had global aphasia at baseline. There was no significant difference between the two groups. The proportions of other types of aphasia were not significantly different between the 2 groups either (Table 4). There were 12 patients in the COT group and 5 patients in the OLT group who had dysphagia, which can affect aphasia. However, there was no significant difference between the two groups (*P* = 0.412). Deafness and dysphonia were not found in these participants.

### 3.4. AQ Score before and after Treatment in the OLT Group

Before language therapy, the total AQ score was 26.7 ± 26.2 in the OLT group. After 7.8 ± 5.7 weeks of conventional therapy with language therapy, the total AQ score increased to 39 ± 31.1, which was a statistically significant change according to the Wilcoxon-signed rank test (*P* = 0.001). All subsections demonstrated improvements in the score and the differences were statistically significant in all subsection scores (Table 5).

### 3.5. AQ Score before and after Treatment in the COT Group

Before language therapy, the total AQ score was 28.1 ± 25.6 in the OLT group. After 6.6 ± 7.1 weeks of oriental medicine therapy combined with language therapy, the total AQ score increased to 45.3 ± 27.4, which was a statistically significant change according to the Wilcoxon signed rank test (*P* < 0.001). All subsections demonstrated improvements in the score and the differences were statistically significant (Table 5).

### 3.6. Comparison of the AQ Score Improvements between the COT and OLT Groups: Total Score and Subsection Scores

The COT group (+17.2 ± 11.6) demonstrated greater improvements in the total AQ score than the OLT group did (+12.3 ± 13.6). This result was statistically significant (*P* = 0.021). The subsections demonstrating better results in the COT group were those for spontaneous speech, content delivery, comprehension, auditory verbal comprehension, command performance, repetition, naming, and naming things. Among them, spontaneous speech, content delivery, comprehension, auditory verbal comprehension, and command performance were statistically significant. The yes-no section exhibited better results in the OLT group with a delta value (value after − value before) of 9 in the OLT group, and 6 in the COT group. However, these differences were not statistically significant (Table 5).

### 3.7. Comparison of the AQ Score Improvements between the COT and OLT Groups: Distribution of Severity

**Figure 2:**
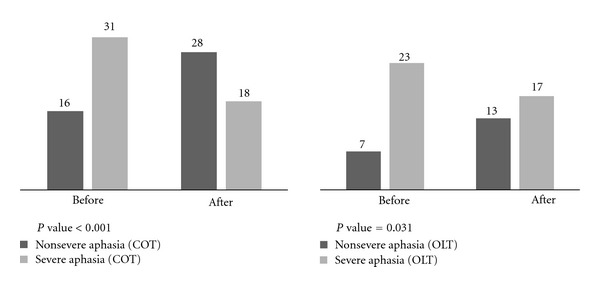
Change of distribution of aphasia severity. COT: group that received language therapy with oriental medicine therapy, OLT: group that received language therapy only, non-Severe: Aphasia Quotient 36.3–92.2, Severe: Aphasia Quotient 0–36.2, *P* value was obtained by the McNemar test for evaluating significance of change of distribution of severe aphasia in each group.

Before language therapy, 66% of patients in the COT group and 76.7% of patients in the OLT group demonstrated severe aphasia. After language therapy, 38.3% of the COT group and 56.7% of the OLT group demonstrated severe aphasia. After language therapy, 41.9% of patients in the COT group and 26.1% of patients in OLT group with severe aphasia were converted into non-severe aphasia. The aphasia subsided in one patient in the COT group. In this change of severe aphasia distribution, the COT group reveals statistically significance. The OLT group also shows statistically significance. Before and after treatment, there was no statistically significant difference between the COT and the OLT group in distribution of severe aphasia (Table 6, Figure 2). Therefore, severe aphasia patients who belonged to the COT group revealed better improvements rather than patients who belonged to the OLT group.

## 4. Discussion

The results of the present study suggest that the administration of language therapy in combination with oriental medicine therapy is effective for the treatment of post-stroke aphasia. Among patients with severe aphasia at onset, those in the COT group exhibited better improvements than those in the OLT group, suggesting that the co-administration of oriental medicine therapy with language therapy revealed more effective tendency in the treatment of patients with severe aphasia. 

The efficacy of language therapy for aphasia has received much attention in recent years. Wertz et al. reported a 6% improvement in patients receiving language therapy compared to those that did not receive conventional language therapy [18]. In a recent meta-analysis, patients who received intense language therapy exhibited a greater improvement than patients who did not receive language therapy [19, 20]. The American BI-ISIG (Brain Injury Interdisciplinary-Specific Interest Group) classified language therapy as a practice standard and an essential item for post-stroke aphasic patients [21]. Therefore, most patients who suffer from post-stroke aphasia usually receive language therapy during their rehabilitation. 

In addition to language therapy, the treatment of patients with aphasia may include pharmacological intervention. Bromocriptine, amphetamines, and piracetam are sometimes used for post-stroke aphasia treatment [9, 10]. The efficacy of these medications has been demonstrated in previous studies. However, it is still controversial whether the improvement of aphasia in patients treated with bromocriptine and amphetamines is caused by an improvement of the aphasia itself or an improvement in their attention. Furthermore, the routine prescription of amphetamines should be avoided because of its potential negative effects on the central nervous system and addiction [22]. Piracetam has been associated with exacerbation of intracerebral hemorrhage and effects on the central nervous system [23]. Therefore, pharmacological interventions have not been used routinely in aphasic patients. In this respect, safe and effective treatments that can be administered in association with language therapy are needed for the treatment of patients with aphasia.

According to previous studies, oriental medicine is an effective therapy for the treatment of the effects of stroke [24–26]. However, the aim of these studies was to evaluate the effects of oriental medicine on the general sequelae of stroke, in particular, motor function disorders. Studies focusing on aphasia were not comparative, but rather case reports or case series [11–17]. The present study was therefore designed to evaluate the effect of the co-administration of oriental medicine with language therapy using a comparative method. 

In addition, the present study assessed the individualized nature of oriental medicine. When Oriental medicine doctors use oriental medicine therapy to treat stroke patients, they adjust the treatment regimen according to each patient's individual needs, general condition, and constitution such as oriental diagnosis. Patients admitted to Oriental medicine hospitals may receive different types of treatment. Therefore, in this study, we used a pragmatic method to investigate the effects of oriental medicine therapy itself on post-stroke aphasia recovery. For example, oriental medicine doctors prescribed *Hyungbangjihwang-tang*, when patients were diagnosed with *Yin deficiency*. 

The present study has following limits. First, because of the study design (non-randomized case-control study), the numbers of participants in each group were not the same. However, there were no significant differences between the two groups in terms of baseline characteristics, type of stroke, type of aphasia, stroke risk factors, and related symptoms. Second, the duration of followup was not the same for all patients. However, there were no significant differences between the 2 groups regarding the duration of language therapy. 

The present data suggest that co-administration of oriental medicine with language therapy can be an effective method for the treatment of post-stroke aphasia. The factors that can affect the improvement of aphasia are first, individual factors such as age, sex, education, and handedness; second, neurological factors such as the severity of aphasia at onset [3], treatment period [3], and cause and sites of brain damage; third, treatment-related factors, including the intensity and duration of language therapy, and the methods used. There were no significant differences in these factors between the two groups in the present study. The exclusion of differences in the factors that affect aphasia recovery between the groups enabled the acquisition of more accurate results. In addition, we believe that this study reflects clinical circumstances more accurately than previous studies. We used a pragmatic method to investigate the effects of oriental medicine therapy itself on post-stroke aphasia recovery. We believe that this method better reflects the individualized character of oriental medicine. Furthermore, we used an objective tool such as the K-WAB to estimate the effect of oriental medicine therapy on post-stroke aphasia. K-WAB is a standardized test for the evaluation of aphasia. The above points demonstrate the reliability and objectivity of the present results. 

A possible mechanism which can explain the result obtained is as follows: oriental medicine therapy affects neural plasticity related to the recovery of language functions such as diaschisis, redundancy, and vicariation [27]. “Diaschisis” means language function that is indirectly damaged has declined for a short period since onset and these functions will be recovered as time passes. “Redundancy” indicates that a language area that is not involved can lead to the recovery of aphasia. Lastly, “vicariation” refers to a brain area that is not in charge of everyday language, but assumes an important role in the recovery from aphasia. Vicariation is assumed a major mechanism of Large artery atherosclerosis (LAA) type stroke patients' aphasia recovery. Vicariation has been considered ineffective because most patients with severe aphasia and extensive brain damage have a tendency to recover slowly. Howevere, we think that a major mechanism of oriental medicine on the recovery of post-stroke aphasia is thought to be vicariation. In this study, 55.3% of COT group patients exhibited an LAA-type ischemic stroke, which was of a higher proportion than in the OLT group. However, the improvement of the COT group was better than that of the OLT group. Furthermore, the COT group exhibited a greater improvement than the OLT group among severely aphasic patients at onset (Figure 2). Therefore, we consider the effect of conventional therapy to be mostly mediated by diaschisis and redundancy and only slightly by vicariation. However, the co-administration of oriental medicine therapy may increase the influence of vicariation. As a result, the COT group exhibited a greater improvement among severely aphasic patients, indicating that oriental therapy can act as a catalyst of vicariation and that it can be effective when it is administered in combination with language therapy.

However, this effect is not limited to the language function area. According to previous studies, oriental medicine can also affect the improvement of motor functions in stroke patients [24]. The results of previous studies showed that oriental medicine can have an effect on neural plasticity related to the recovery of motor function. Together with the present results, these studies suggest that oriental medicine can affect brain neural plasticity in relation to both language and motor functions, and the improvement of aphasia may be the result of the activation of whole-brain neural plasticity. Future studies should assess the recovery of language disorders together with other stroke symptoms to validate this hypothesis.

## 5. Conclusion

The present study assessed the effect of co-administration of oriental medicine therapy with language therapy on post-stroke aphasia 6 months before stroke onset. This study was conducted as a comparative, observational, and pragmatic study. The results of this study are as follows.

1. The COT group exhibited a greater improvement than the OLT group in the total AQ score and most subsection scores. In particular, there were statistically significant differences in total AQ score and subsections such as spontaneous speech, content delivery, comprehension, auditory verbal comprehension, and command performance.
2. Among severe aphasic patients, the improvement of the COT group was better than that of the OLT group There was statistically significant differences in distribution of severe aphasia of the COT group. However, there were no statistically significance in the OLT group.

Therefore, we suggest that combination therapy with the administration of oriental medicine therapies and language therapy can be helpful in the treatment of post-stroke aphasic patients 6 months before stroke onset.

## Figures and Tables

**Figure 1:**
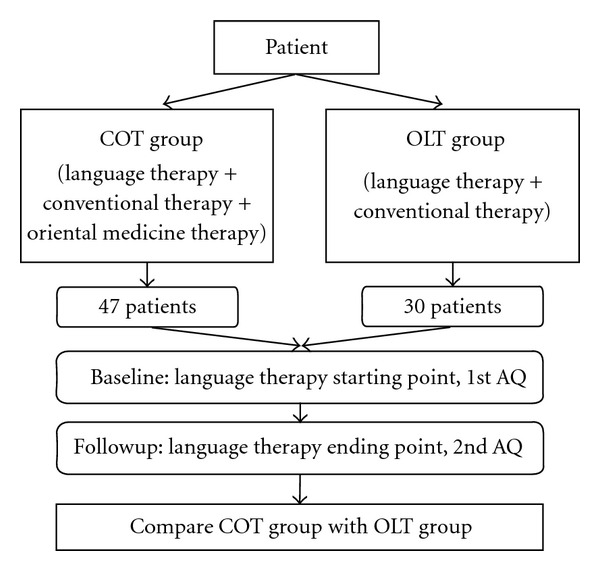
Study protocol.

**Table 1:** Oriental medicine therapies referred to COT group.

Therapy	Contents		Proportion (%)
	*Cheongshinhaeeo-tang*	Arisaematis Rhizoma, Pinelliae Rhizoma 8 Saposhnikovia Radix, Poria, Paeoniae Radix Alba, Rehmanniae Radix, Acori Graminei Rhizoma, Linderae Radix, Polygalae Radix, Aurantii Immaturus, Citri Pericarpium, Ligustici Rhizoma, Coptidis Rhizoma, Angelicae Gigantis Radix, Liriopes Radix, Glycyrrhizae Radix, and Notopterygii Rhizoma 4	21 (44.7)
	*Jasoohaeeo-tang*	Glycyrrhizae Radix, Notopterygii Rhizoma 6, Cinnamomi Ramulus, Gazellae Cornu 8, Saposhnikovia Radix, Pulvis Aconiti Tuberis Purificatum, Zizyphi Spinosae Semen, Gastrodiae Rhizoma 4	3 (6.4)
	*Hyungbangjihwang-tang*	Poria, Alismatis Rhizoma, Corni Fructus, Rehmanniae Radix Preparata 16 Notopterygii Rhizoma, Angelicae Pubescentis Radix, Schizonepeta Spica, Saposhnikovia Radix, and Plantaginis Semen 8	6 (12.8)
Herbal medicine (g/day)	*Yeoldahanso-tang*	Puerariae Radix 32 Ligustici Sinense Radix, Scutellariae Radix 16 Platycodi Radix, Raphani Semen, Angelicae Dahuricae Radix, and Cimicifugae Rhizoma 8	8 (17)
	*Seonghyangjeonggi-san*	Pogostemi Herba 12 Platycodi Radix, Arecae Pericarpium, Perillae Folium 8 Citri Pericarpium, Magnoliae Cortex, Aucklandiae Radix, Pinelliae Rhizoma, Poria, Angelicae Dahuricae Radix, Atractylodis Rhizoma Alba, Arisaematis Rhizoma, and Glycyrrhizae Radix 4	3 (6.4)
	*Bojoongikgi-tang*	Astragali Radix 12 Glycyrrhizae Radix, Atractylodis Rhizoma Alba, Ginseng Radix 8 Angelicae Gigantis Radix, Citri Pericarpium 4 Cimicifuga Rhizoma, and Bupleuri Radix 2	6 (12.8)

	LI4, LI11, GB20, LR3 (both sides), LI10, ST36, ST37, TE3, GB34,		47 (100)
Acupuncture (1 time/day)	GB39, GB41, TE5 (affected side), HT7, PC6 (nonaffected side),		
	GV15, GV20, and GV23		

Electroacupuncture (1 time/day)	LI4, LI10, LI11, ST36, ST37, GB39, GB41, and TE5 (affected side)		47 (100)

Moxibustion (1 time/day)	CV4 and CV12		47 (100)

COT group: group that received language therapy with oriental medicine therapy.

**Table 2:** Patient characteristics in the 2 groups.

	COT group (*n* = 47)	OLT group (*n* = 30)	*P*-value
Gender, male (%)	31 (66)	14 (46.7)	0.104
Age, yr (SD, median)	54.5 (12.1, 54)	52.5 (17.6, 55.5)	0.790
Education, yr (SD, median)	10.8 (5.1, 12)	11.2 (3.8, 12)	0.918
Handedness, right (%)	46 (97.9)	30 (100)	1.000
Treatment period from onset, day (SD, median)	48 (45.4, 29)	41 (28.7, 39)	0.954
Duration of treatment, weeks (SD, median)	6.6 (7.1, 4.9)	7.8 (5.7, 6.4)	0.104
Hypertension (%)	31 (65.96)	20 (66.67)	1.000
Diabetes mellitus (%)	12 (25.53)	3 (10)	0.140
Dyslipidemia (%)	8 (17.02)	3 (10)	0.513
Heart disease (%)	7 (14.89)	4 (13.33)	1.000
AQ scores (SD, median)	28.1 (25.6, 19.2)	26.7 (26.2, 19)	0.875
Total score	5.5 (5.1, 3)	5.7 (5.4, 5)	0.757
*Spontaneous speech *	3.3 (2.8, 2)	3.4 (3, 3)	0.987
Content delivery	2.2 (2.4, 1)	2.5 (2.8, 1.5)	0.928
Fluency	3.6 (2.9, 3.2)	3.6 (2.7, 3.1)	0.979
*Comprehension *	32.1 (17.9, 33)	29.6 (18.9, 32)	0.645
Yes-no	21.1 (20.9, 11)	21.9 (20.4, 18.5)	0.892
Auditory verbal comprehension	19.8 (23.4, 10)	19.7 (21.4, 12)	0.834
Command performance	3.3 (3.6, 1.2)	2.4 (3.4, 0.6)	0.179
*Repetition *	1.9 (2.6, 0.5)	1.6 (2.6, 0.2)	0.405
*Naming *	12.9 (17.9, 4)	11.4 (18.1, 0.5)	0.457
Naming things	1.2 (2.8, 0)	1.2 (2.5, 0)	0.510
Control word association	2.5 (3.6, 0)	2 (3.3, 0)	0.472
Sentence completion	1.9 (3.3, 0)	1.7 (3.3, 0)	0.670
Sentence answers			

COT group: group that received language therapy with oriental medicine therapy.

OLT group: group that received language therapy only.

AQ: aphasia Quotient.

*P* value was obtained by the Mann-Whitney *U* test (age, education, treatment period from onset, duration of treatment, and AQ scores) and Fisher's exact test (gender and handedness).

SD: standard deviation.

**Table 3:** Type of stroke.

	COT group (*n* = 47)	OLT group (*n* = 30)	*P* value
Infarction : hemorrhage	30 : 17	14 : 16	
Infarction			
LAA (%)	26 (55.32)	10 (33.33)	0.670
CE (%)	2 (4.26)	1 (3.33)	1.000
SAO (%)	0 (0)	1 (3.33)	0.390
Other causes (%)	1 (2.13)	1 (3.33)	1.000
Undetermined causes (%)	1 (2.13)	0 (0)	1.000
Hemorrhage			
ICH (%)	13 (27.66)	13 (43.33)	0.217
IVH (%)	1 (2.13)	0 (0)	1.000
SDH (%)	0 (0)	2 (6.67)	0.149
SAH (%)	3 (6.39)	1 (3.33)	1.000

COT group: group that received language therapy with oriental medicine therapy.

OLT group: group that received language therapy only.

LAA: large artery atherosclerosis. CE: cardioembolism.

SAO: small artery occlusion. ICH: intracerebral hemorrhage.

IVH: intraventricular hemorrhage. SDH: subdural hemorrhage.

SAH: subarachnoid hemorrhage.

*P* value was obtained by Fisher's exact test.

**Table 4:** Type of aphasia at time of admission.

	COT group (*n* = 47)	OLT group (*n* = 30)	*P* value
Motor (%)	7 (14.89)	6 (20)	0.756
Sensory (%)	3 (6.38)	3 (10)	0.673
Global (%)	25 (53.19)	16 (53.33)	1.000
Conduction (%)	1 (2.13)	0 (0)	1.000
Transcortical (%)	7 (14.89)	2 (6.67)	0.469
Anomic (%)	4 (8.51)	3 (10)	1.000

COT group: group that received language therapy with oriental medicine therapy.

OLT group: group that received language therapy only.

*P* value was evaluated by Fisher's exact test.

**Table 5:** AQ scores before and after language treatment in the OLT and COT group.

AQ	OLT group (SD, median)			COT group (SD, median)			*P* value*
	Before	After	Delta value	Before	After	Delta value
Total score	26.7 (26.2, 19)	39^#^ (31.1, 31.6)	12.3 (13.6, 7.2)	28.1 (25.6, 19.2)	45.3^#^ (27.4, 41.3)	17.2 (11.6, 16.9)	0.021
Spontaneous speech	5.7 (5.4, 5)	8.2^#^ (6.4, 6.8)	2.5 (3.4, 1.5)	5.5 (5.1, 3)	9^#^ (5.4, 8.5)	3.5 (2.9, 3)	0.049
Content delivery	3.4 (3, 3)	4.6^#^ (3.5, 4)	1.2 (2.1, 1)	3.3 (2.8, 2)	5.2^#^ (2.7, 5)	1.9 (1.7, 2)	0.024
Fluency	2.5 (2.8, 1.5)	3.7^#^ (3.3, 2.3)	1.2 (1.9, 1)	2.2 (2.4, 1)	3.9^#^ (4.4, 6.2)	1.8 (1.7, 1)	0.097
Comprehension	3.6 (2.7, 3.1)	4.8^#^ (3, 4.4)	1.3 (1.4, 0.9)	3.6 (2.9, 3.2)	6.2^#^ (4.4, 6.2)	2.7 (4.3, 1.9)	0.009
Yes-no	29.6 (18.9, 32)	35.7^#^ (19.3, 37.5)	7.9 (10.4, 9)	32.1 (17.9, 33)	43^#^ (15.6, 48)	11 (13.1, 6)	0.574
Auditory verbal comprehension	21.9 (20.4, 18.5)	29.8^#^ (21.3, 26.5)	8 (9.3, 4)	21.1 (20.1, 11)	36.2^#^ (19.8, 36)	15.1 (13.7, 12)	0.019
Command performance	19.7 (21.4, 12)	28.2^#^ (26, 21.5)	8.7 (15.6, 1.5)	19.8 (23.4, 10)	34.1^#^ (26, 32)	14.2 (15.5, 8)	0.031
Repetition	2.4 (3.4, 0.6)	3.6^#^ (3.7, 2.6)	1.3 (1.8, 0.5)	3.3 (3.6, 1.2)	5.5^#^ (5.3, 5.2)	2.1 (3.9, 1.3)	0.100
Naming	1.6 (2.6, 0.2)	2.8^#^ (3.2, 1.2)	1.2 (1.5, 0.8)	1.9 (2.6, 0.5)	3.4^#^ (3, 2.1)	1.5 (1.7, 1.2)	0.274
Naming things	11.4 (18.1, 0.5)	19.3^#^ (21.1, 9.5)	8 (11, 4.5)	12.9 (17.9, 4)	21.1^#^ (19.2, 14)	8.2 (10.8, 6)	0.626
Control word association	1.2 (2.5, 0)	3.1^#^ (4.6, 0)	1.9 (2.9, 0)	1.2 (2.8, 0)	2.7^#^ (4.2, 0)	1.5 (3.4, 0)	0.427
Sentence completion	2 (3.3, 0)	2.9^#^ (3.8, 1)	0.9 (1.8, 0)	2.5 (3.6, 0)	4^#^ (3.7, 4)	1.5 (2.5, 0)	0.391
Sentence answer	1.7 (3.3, 0)	2^#^ (4.1, 0)	1 (2.3, 0)	1.9 (3.3, 0)	3.3^#^ (3.8, 2)	1.5 (2.7, 0)	0.215

COT group: group that received language therapy with oriental medicine therapy.

OLT group: group that received language therapy only.

*P value was obtained by the Mann-Whitney *U* test for comparing delta values between the OLT and the COT group.

^#^ *P* value means “<0.05.” It was obtained by the Wilcoxon-signed rank test for evaluating significance of change in each group.

**Table 6:** Comparison of the AQ Score improvements between the COT and OLT groups: distribution of severity.

Group	Aphasia severity	COT group (*n* = 47)	OLT group (*n* = 30)	*P* value
Before treatment (%)	Severe	31 (66)	23 (76.7)	0.445
	Nonsevere	16 (34)	7 (23.3)	

After treatment (%)	Severe	18 (38.3)	17 (56.7)	0.159
	Non-severe	29 (61.7)	13 (43.3)	

COT group: group that received language therapy with oriental medicine therapy.

OLT group: group that received language therapy only.

*P* value was obtained by Fisher's exact test for evaluating significance of distribution of severe aphasia in each point (before treatment and after treatment).

**Table 7:** AQ (Aphasia Quotient) test subsection.

Spontaneous speech
Content delivery
Fluency
Comprehension
Yes-no
Auditory verbal comprehension
Command performance
Repetition
Naming
Naming things
Control word association
Sentence completion
Sentence answer

Total AQ score = spontaneous speech + comprehension + repetition + naming.
